# Assessment of Placental Antioxidant Defense Markers in Vaccinated and Unvaccinated COVID-19 Third-Trimester Pregnancies

**DOI:** 10.3390/life14121571

**Published:** 2024-11-29

**Authors:** Alessandro Rolfo, Stefano Cosma, Anna Maria Nuzzo, Laura Moretti, Annalisa Tancredi, Stefano Canosa, Alberto Revelli, Chiara Benedetto

**Affiliations:** 1 Department of Surgical Sciences, Gynecology and Obstetrics 2, City of Health and Science—S. Anna University Hospital, University of Turin, 10126 Turin, Italy; a.nuzzo@unito.it (A.M.N.); l.moretti@unito.it (L.M.); alberto.revelli@unito.it (A.R.); 2 Department of Surgical Sciences, Gynecology and Obstetrics 1, City of Health and Science—S. Anna University Hospital, University of Turin, 10126 Turin, Italy; stefano.cosma@unito.it (S.C.); annalisa.tancredi@unito.it (A.T.); s.canosa88@gmail.com (S.C.); chiara.benedetto@unito.it (C.B.)

**Keywords:** COVID-19, SARS-CoV-2 vaccination, pregnancy, placenta, oxidative stress

## Abstract

Background: Pregnancy has been identified as a risk factor for severe COVID-19, leading to maternal and neonatal complications. The safety and effects of the SARS-CoV-2 vaccination during pregnancy, particularly on placental function and oxidative stress (OxS), remain underexplored. We investigated the impact of vaccination on third-trimester placental antioxidant defense markers. Methods: Ninety full-term pregnant women were divided into the following groups: vaccinated (n = 27) and unvaccinated (n = 25) COVID-19-positive pregnant women; control subgroups were composed of vaccinated (n = 19) or unvaccinated (n = 19) COVID-19-negative women with a healthy term singleton pregnancy with no signs of COVID-19. Placental samples were collected after delivery. Lipid peroxidation (TBARS), gene expression of HIF-1α, and catalase (CAT), superoxide dismutase-1 (SOD1) and CAT-SOD1 enzymatic activity were measured. Results: COVID-19-positive placentae exhibited significantly higher TBARS and HIF-1α levels compared to controls, regardless of vaccination status. Vaccination significantly increased placental CAT and SOD1 expression and activity in COVID-19-positive women, suggesting enhanced antioxidant defense. Unvaccinated women showed a higher incidence of COVID-19 symptoms and lower antioxidant enzyme activity. Conclusions: SARS-CoV-2 infection induced placental OxS, which is countered by a placental adaptive antioxidant response. Vaccination during pregnancy enhances placental defense, further supporting the safety and benefits of COVID-19 vaccination in preventing complications and protecting fetal development.

## 1. Introduction

The Coronavirus Disease 2019 (COVID-19) pandemic, with more than six million deaths worldwide, has been a major source of concern over the past years. Pregnancy has been identified as an independent risk factor for severe COVID-19 disease, with implications for both the mother and the fetus [1]. Several studies have demonstrated increased rates of intensive care, invasive ventilation, extracorporeal membrane oxygenation, pre-eclampsia, premature delivery, stillbirth, neonatal and maternal mortality [2,3], that have been observed even in healthy women with no co-morbidities [4].

In late 2020, Severe Acute Respiratory Syndrome Coronavirus 2 (SARS-CoV-2) vaccines were introduced, resulting in a reduction of more than 85% in the risk of developing symptomatic disease and transmitting the virus [5]. Although the vaccines were not tested in pregnancy, the American College of Obstetricians and Gynecologists (ACOG) [6] recommended their use for pregnant women. Pregnancy-specific concerns included the potential effects of vaccination on maternal safety, fetal development and placental physiopathology.

Despite the fact that pregnancy was an exclusion criterion in early SARS-CoV-2 vaccine trials, observational data were quickly collected, demonstrating that the benefits of vaccination outweighed its potential risks, and emphasizing vaccine safety in pregnancy, its protective effect against symptomatic disease, and minimal adverse fetal outcomes [7,8,9,10]. Key findings in favor of SARS-CoV-2 vaccine safety were that there were no signs of the mRNA vaccine in the maternal blood, placenta or cord blood at delivery, and that maternal IgM was not detected in the umbilical cord blood following vaccination in pregnancy [11,12,13], indicating that the vaccine did not cross the placental barrier and did not elicit an immune response in the fetus. As previously demonstrated for pertussis, tetanus, and influenza [14,15,16], maternal IgG raised by SARS-CoV-2 vaccination crossed the placenta and was found in the umbilical cord blood at birth [11,12,13,17,18,19,20]. Overall, these findings indicated that a direct effect of vaccination on fetal development was unlikely. This was also confirmed by several authors whose studies showed no evidence of an increased risk of preterm birth, small size for gestational age, or stillbirth after SARS-CoV-2 vaccination [7,8,10,21,22,23]. In line with these data, the European Medicine Agency (EMA) COVID-19 task force (ETF) also reviewed several studies involving approximately 65,000 pregnancies at different stages, failing to find any sign of an increased risk of obstetrical complications, miscarriages, preterm births or adverse fetal effects following SARS-CoV-2 vaccination [24]. In a population-based cohort study conducted to assess neonatal safety following the COVID-19 mRNA vaccination during pregnancy, which included 196,470 newborns in Sweden and Norway, the findings indicated that infants that had pre-natal exposure to vaccination showed statistically lower odds of neonatal intracranial hemorrhage, hypoxic-ischemic encephalopathy and mortality, while there was no significant increase in adverse outcomes [25]. These findings further contribute to the evidence supporting the COVID-19 mRNA vaccine’s safety during pregnancy. It must be remembered that SARS-CoV-2 vaccination is specifically endorsed to protect pregnant women from severe illness, and is advised for specific high-risk conditions, like hepatitis and meningococcal disease [26].

While vascular pathological changes associated with COVID-19 infection, such as thrombosis, malperfusion and vasculopathy, have been extensively reported on both maternal and fetal–placental sides [27], a few studies have specifically examined the placental response to SARS-CoV-2 vaccination. Morphological studies have demonstrated that vaccination did not cause placental abnormalities such as intervillositis, trophoblast necrosis, increased fibrin or MPFD (massive perivillous fibrin/fibrinoid deposition), villitis or thrombohematomas [28]. Moreover, correlations between clinical and epidemiologic data and those from placental pathology studies have suggested that a potential (and even likely) mechanism of fetal protection from COVID-19 infection could arise from maternal vaccination, preventing maternal viremia, placental infection and vasculopathy [27,29].

SARS-CoV-2 vaccination efficacy is known to be strictly dependent on the maternal immune response, and several exogenous and endogenous factors can interfere [30]. A relevant role is played by the Oxidative State (OxS), determined by the balance between pro-oxidant and antioxidant factors [30]. In this context, our group recently demonstrated increased OxS markers of TBARS and HIF-1α in the placenta of women infected with COVID-19 during the third trimester of pregnancy, accompanied by increased placental antioxidant superoxide-dismutase (SOD) and catalase (CAT) enzymatic activity, suggesting a compensatory antioxidant defense adaptation aimed at protecting placental physiology and fetal growth [31]. Our previous findings indicated a placental OxS inhibition in response to COVID-19 that was able to counteract the viral cytotoxic activity. To this regard, however, nothing is known about the effect of SARS-CoV-2 vaccination on the placental response to OxS in pregnant women.

In the present study, we investigated the placental antioxidant asset, involving CAT and SOD enzymes, comparing (a) vaccinated vs. unvaccinated COVID-19-positive women during the third trimester of pregnancy, and (b) vaccinated or unvaccinated COVID-19-positive pregnant women vs. vaccinated or unvaccinated COVID-19-negative pregnant women.

## 2. Materials and Methods

### 2.1. Study Population and Tissue Collection

A total number of 90 full-term pregnant women were recruited at the Gynecology and Obstetrics 1 and 2 Units, Sant’Anna University Hospital, City of Health and Science, Turin (Italy), between 1 January and 31 March 2022. Fifty-two of them were identified as positive for COVID-19 with a nasopharyngeal swab, with the virus detected by a reverse transcriptase-polymerase chain reaction (RT-PCR) assay (Liferiver Bio-Tech, San Diego, CA, USA). These 52 COVID-19-positive pregnant women were divided into two subgroups: vaccinated (v-COVID-19; n = 27), who received at least 1 dose of the SARS-CoV-2 mRNA vaccine (Pfizer-BioNTech, Collegeville, PA, USA; or ModernaTX, Cambridge, MA, USA); and unvaccinated (u-COVID-19; n = 25), who did not receive any SARS-CoV-2 vaccine before delivery. The control subgroups were composed of vaccinated (v-CTRL; n = 19) or unvaccinated (u-CTRL; n = 19) COVID-19-negative women with a healthy term singleton pregnancy, with no signs of maternal, placental or fetal COVID-19 disease.

After delivery, four full-thickness biopsies were randomly collected from the placental basal plate (intermediate area) and immediately frozen. Calcified, necrotic and seriously damaged areas were excluded. The samples were subsequently processed for mRNA and protein isolation. Maternal anamnesis and neonatal outcome data were recorded.

### 2.2. Assessment of OxS Markers

Since ROS are highly reactive and have a very short half-life, their direct detection in tissue and body fluids with accuracy and precision is unfeasible [32]. Alternatively, a valid method to detect OxS in biological samples is to investigate ROS-mediated oxidative damage to cell lipids, proteins and nucleic acids [33]. Herein, placenta plasma membrane lipid peroxidation was estimated by determining the Thiobarbituric Acid Reactive Substances (TBARS) using a TBARS Assay Kit (Cayman chemical, Ann Arbor, MI, USA). Absorbance was measured at 535 nM on a microplate reader and TBARS values were calculated using a Malondialdehyde (MDA) standard curve, prepared by acid hydrolysis of 1,1,3,3tetramethoxypropane. The values were expressed as MDA uM.

### 2.3. Placental RNA Isolation and Real-Time PCR

TRI^®^ reagent (Sigma-Aldrich, Milan, Italy) was used to isolate total placental RNA from frozen biopsies following the manufacturer’s instructions. DNAse I was used to remove genomic DNA contaminations. 3 µg of total RNA was reverse-transcribed using a random hexamers approach (Fermentas Europe, St. Leon-Rot, Germany, Cat. No k1632). Hypoxia-Inducible Factor-1α (HIF-1α), CAT, and SOD1 gene expression levels were determined by real-time PCR using specific TaqMan primers and probes (Life Technologies, Carlsbad, CA, USA, Cat. No 4331182). Ribosomal 18S RNA expression was used as an internal reference (Life Technologies, Cat. No 4333760F) and relative expression was calculated according to Livak and Schmittgen [34].

### 2.4. CAT and SOD Enzymatic Activity

Placental CAT and SOD enzymatic activity were assessed using commercially available kits (Cayman Chemical, Ann Arbor, MI, USA). In brief, CAT peroxidative function was measured based on the reaction between CAT and methanol in the presence of hydrogen peroxide of optimal concentration. 4-amino-3-hydrazino-5-mercapto-1,2,4-triazole was used to spectrophotometrically quantify formaldehyde at 540 nm. The results were expressed in nmol/min/mL. Total SOD activity was measured with cytochrome C reduction by superoxide (O2^•−^) radicals monitored spectrophotometrically at 450 nm, using the xanthine-xanthine oxidase system. The results were expressed in U/mL.

### 2.5. Statistical Analysis

As the data did not show a normal distribution, the Kruskal–Wallis non-parametric test and the Mann–Whitney U-test with Bonferroni’s correction were performed. The data are presented as Mean ± SE (standard error). The categorical variables are presented as percentages; the comparison between groups was performed using a chi-square test.

SPSS Version 29 statistical software (IBM Corp., Chicago, IL, USA) was used for statistical analysis. The significance was set at *p* < 0.05.

## 3. Results

### 3.1. Clinical Features of the Study Population

A total of 52 pregnant women tested positive for COVID-19 infection at delivery; among them, 27 were included in the v-COVID-19 subgroup as they received at least one dose of a SARS-CoV-2 mRNA vaccine; only 8 of them (29.6%) completed the vaccination course of three doses, while 16 (59.2%) received two doses and 3 (11.1%) received one dose. The average time between the last dose of the SARS-CoV-2 vaccine and delivery was 103.1 ± 18.6 days (range 14–377 days), and 83.3% of the v-COVID-19 patients received the last dose during pregnancy. The other 25 COVID-19-positive patients were included in the u-COVID-19 subgroup, as they did not receive any SARS-CoV-2 vaccine.

The control (CTRL) subgroups were composed of 38 pregnant women who tested negative for COVID-19 infection at delivery; they were categorized as vaccinated (v-CTRL; n = 19) or unvaccinated (u-CTRL; n = 19). In the v-CTRL group, 10 (52.6%) completed the vaccination course, 8 (42.1%) received two SARS-CoV-2 vaccine doses and 5.3% received only one dose.

The clinical features of the study population are presented in Table 1. Maternal age, gestational age at delivery, nulliparous percentage and cesarean section rate were comparable among groups. Pre-pregnancy co-morbidity did not significantly differ among the subgroups, nor was the rate of obstetrical complications significantly different. The COVID-19-positive groups presented no stillbirths and they did not require neonatal respiratory support within 24 h of birth. Among the COVID-19-positive women, symptoms like fever and anosmia, ageusia and asthenia were more frequently observed in unvaccinated women (32% and 16%, respectively) than in vaccinated women (7.4% and 0%, respectively).

### 3.2. OxS Markers TBARS and HIF-1α in v-COVID-19 and u-COVID-19 Placentae

**Figure 1:**
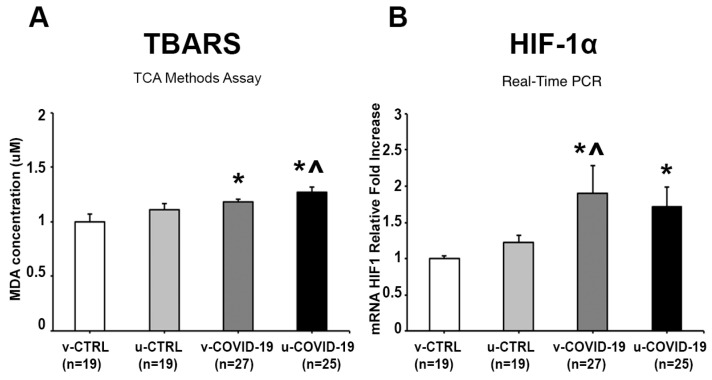
Effects of COVID-19 infection on oxidative stress markers in the placentae of the v-COVID-19, u-COVID-19, v-CTRL and u-CTRL subgroups: (**A**) TBARS and (**B**) HIF-1α gene expression. * significant vs. v-CTRL; ^ significant vs. u-CTRL.

We found a significant TBARS increase in the v-COVID-19 (*p* = 0.05, 1.2-fold increase) and u-COVID-19 (*p* = 0.01, 1.3-fold increase) placentae compared to the v-CTRL subgroup, and in the u-COVID-19 vs. u-CTRL placentae (*p* = 0.05, 1.14-fold increase) (Figure 1A). No significant differences were reported between the v-COVID-19 and u-COVID-19 subgroups (*p* > 0.05) (Figure 1A). Overall, these findings suggested the presence of a significantly increased placental OxS linked to COVID-19 infection.

The gene expression level of hypoxia-inducible factors-1α (HIF-1α), a main player in the cellular response to hypoxia and OxS, was significantly increased in the v-COVID-19 (*p* = 0.04, 1.9-fold increase) and u-COVID-19 (*p* = 0.019, 1.2-fold increase) placentae compared to the v-CTRL subgroup (Figure 1B), and in the v-COVID-19 subgroup relative to the u-CTRL subgroup (*p* = 0.02, 1.6-fold increase) (Figure 1B). No significant differences were reported between the v-COVID-19 and u-COVID-19 groups (*p* > 0.05) (Figure 1B).

### 3.3. Evaluation of Placental Antioxidant Markers

**Figure 2:**
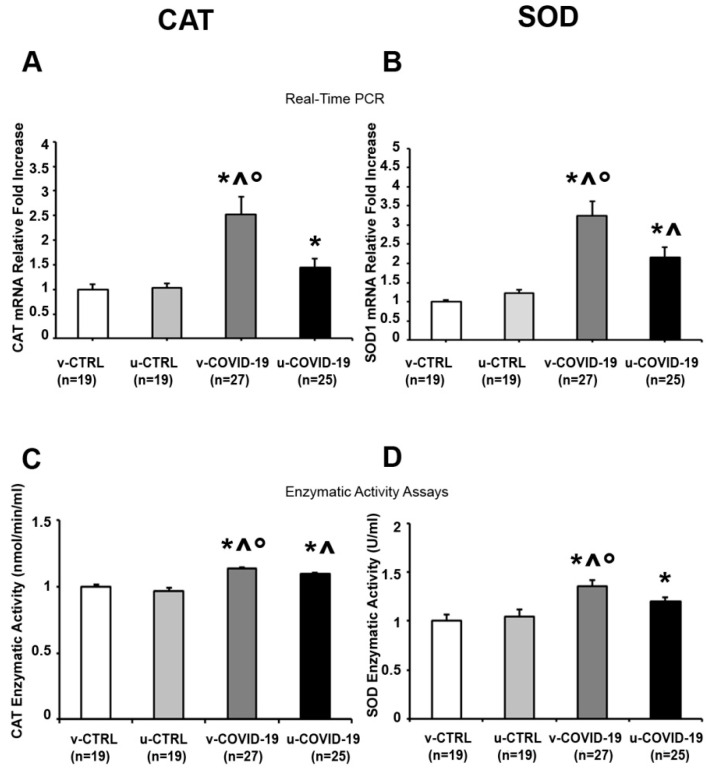
Effects of COVID-19 infection on antioxidant defense markers in the placenta from the v-COVID-19, u-COVID-19, v-CTRL and u-CTRL group: (**A**) mRNA expression and (**C**) enzymatic activities of CAT, (**B**) mRNA expression and (**D**) enzymatic activity of SOD. * significant effect vs. v-CTRL; ^ significant effect vs. u-CTRL; ° significant effect vs. u-COVID-19.

Antioxidant enzymes prevent OxS-mediated tissue damage by detoxifying free radicals. SOD1 catalyzes the conversion of the O2^●−^ radical to H_2_O_2_; then, cytosolic CAT transforms H_2_O_2_ into water. We reported significantly higher CAT mRNA levels in the placentae of the v-COVID-19 (*p* = 0.009, 2.5-fold increase) and u-COVID-19 (*p* = 0.04, 1.4-fold increase) subgroups compared to the v-CTRL subgroup (Figure 2A). Moreover, the v-COVID-19 placentae significantly over-expressed the CAT gene in comparison with the u-CTRL placentae (*p* = 0.007, 2.4-fold increase). Importantly, a significant increase in placental CAT expression was reported in the v-COVID-19 relative to the u-COVID-19 subgroup (*p* = 0.034, 1.5-fold increase). In line with these data, placental CAT enzymatic activity was significantly increased in the v-COVID-19 and u-COVID-19 subgroups compared to the v-CTRL (*p* < 0.001, 1.1-fold increase; *p* < 0.001, 1.1-fold increase) and u-CTRL subgroups (*p* = 0.002, 1.2-fold increase; *p* = 0.001, 1.1-fold increase) (Figure 2C). Importantly, CAT enzymatic activity was significantly increased in the v-COVID-19 placentae relative to the u-COVID-19 placentae (*p* = 0.017, 1.1-fold increase).

CAT overexpression was accompanied by a significant increase in SOD1 mRNA levels in v-COVID-19 (*p* < 0.001, 3.2-fold increase; *p* = 0.001, 2.7-fold increase) and u-COVID-19 (*p* < 0.001, 2.1-fold increase; *p* = 0.017, 1.8-fold increase) placentae compared to the v-CTRL and u-CTRL subgroups (Figure 2B). Moreover, SOD1 gene expression levels were significantly over-expressed in the v-COVID-19 placentae relative to the u-COVID-19 placentae (*p* = 0.04, 1.5-fold increase). SOD1 gene overexpression was accompanied by a significant increase in SOD enzymatic activity in the v-COVID-19 (*p* = 0.007, 1.3-fold increase) and u-COVID-19 (*p* = 0.002, 1.2-fold increase) subgroups compared to the v-CTRL subgroup (Figure 2D). Moreover, SOD enzymatic activity was significantly increased in the v-COVID-19 placentae relative to the u-CTRL placentae (*p* = 0.001, 1.3-fold increase) and, importantly, the u-COVID-19 (*p* = 0.04, 1.1-fold increase) subgroup.

## 4. Discussion

Despite the exclusion of pregnant women from initial phase 3 clinical trials [7], it is now well known that SARS-CoV-2 vaccination is safe in pregnancy and represents the most important intervention to protect gestation from COVID-19-related morbidity and mortality, in the meantime offering protection to the newborn at birth [35]. Most previous studies on the impact of SARS-CoV-2 vaccination during pregnancy, however, have focused on maternal and infant outcomes, without considering the possible effects on the placental tissue. Herein, in order to better understand the consequences of SARS-CoV-2 vaccination on pregnancy and fetal–placental development, we investigated obstetrical and neonatal outcomes together with placental OxS molecular markers and antioxidant activity. In accordance with our previously published data [31], we confirmed a significant increase in both the OxS markers TBARS and HIF-1α and the antioxidant CAT and SOD1 enzymes in COVID-19-positive placentae compared to COVID-19-negative controls, emphasizing the placental ability to counteract COVID-19-induced OxS during pregnancy. For the first time, we also demonstrated that SARS-CoV-2 vaccination significantly increased the antioxidant enzymes CAT and SOD1 in the placentae of COVID-19-positive patients, eliciting a boost in placental protection against OxS.

From a clinical perspective, we reported herein a significantly increased incidence of COVID-19-related maternal symptoms in unvaccinated vs. vaccinated pregnant women who were positive for the virus at delivery. Our findings are in line with previous data showing that pregnant women who received at least one dose of the vaccine before COVID-19 infection had an 80% lower risk of developing severe symptoms [36]. In our study, the increased incidence of maternal symptoms in unvaccinated COVID-19-positive pregnant women was associated with a lower placental antioxidant defense, suggesting a reduced placental compensatory adaptation despite no significant changes in neonatal birth weight and/or Apgar score. While this study focused on molecular and biochemical markers of oxidative stress, collaboration with a pathologist for a detailed placental histopathological examination could have provided additional insights into potential structural changes or localized effects that might not be evident through molecular markers alone. Future studies incorporating such analyses may help to provide a more comprehensive understanding of placental responses to SARS-CoV-2 infection and vaccination.

Our results are in line with those of Smithgall et al., who also reported no differences in placental anatomopathological findings when comparing the placenta of 164 vaccinated and 267 unvaccinated women [37].

Exacerbated inflammation and OxS have been proposed as key players in COVID-19 pathophysiology [31,38,39]. Indeed, we observed a significant increase in placental lipid peroxidation and HIF-1α expression in all COVID-19-positive women. COVID-19 infection, in fact, compromises mitochondrial structure and functionality, triggering the production of reactive oxygen species (ROS) [31] as well as an inflammatory response with the release of cytokines as interleukin-10 (IL-10), tumor necrosis factor-alpha (TNF-α) and interferon-gamma (INF-γ). This inflammatory pathway further reinforces mitochondrial ROS production and OxS [39,40]. Besides being culprits of COVID-19 infection, ROS production and OxS could also be generated as a consequence of immunization and antibody production by activated B and T immune cells, as suggested by observations of increased ROS levels after the first dose of mRNA-based SARS-CoV-2 vaccines [41].

The first line of defense against OxS consists in the overproduction of the antioxidant enzymes CAT and SOD1, which convert superoxide radicals into hydrogen peroxide and then into water plus molecular oxygen [42]. In the present study, a significant increase in CAT and SOD1 expression and activity was observed in vaccinated COVID-19-positive placentae compared to both unvaccinated and control COVID-19-negative patients. Recently, IgGs carrying CAT activity were found to be significantly increased in non-pregnant patients who had recovered from COVID-19 compared to both healthy women and patients vaccinated with the recombinant Sputnik V vaccine, opening up the hypothesis that COVID-19 infection may stimulate the production of antibodies with enzymatic activity that degrades hydrogen peroxide and counteracts ROS production [43]. The increased CAT expression and activity that we observed in the v-COVID-19 placentae suggests that vaccination further increases the ability to scavenge hydrogen peroxide; this could represent an adaptive response to the immunization, aimed at neutralizing the effect of ROS and preventing membrane lipid peroxidation.

Besides promoting inflammation and oxidative injury, increased ROS levels are able to further stimulate viral replication [44,45]. CAT is the most abundant and effective catalyst for the decomposition of H_2_O_2_ [46], being able to breakdown 107 H_2_O_2_ molecules in 1 s. Based on this, Qin et al. recently explored the effectiveness of CAT administration as a potential treatment for COVID-19, showing that the administration of nano-encapsulated CAT to Rhesus macaques elicited cytoprotection, increased the viability of HPAEpiC human pulmonary alveolar epithelial cells, down-regulated the leukocyte production of TNF-α and IL-10, and inhibited COVID-19 replication, without noticeable toxicity [47]. These data underline the therapeutic potential of exogenous CAT administration, further emphasizing the importance of endogenous placental CAT overactivation promoted by anti-SARS-CoV-2 vaccination.

In mice immunized through the administration of recombinant SARS-CoV-2 spike protein antigens, the decline in anti-COVID-19 antibodies caused by OxS and SOD decreased upon virus re-exposure, and the activation of the JAK2/STAT1 pathway was efficiently counteracted by upregulating SOD production [48]. These findings suggest that the vaccination-related boost in placental SOD expression and activity described in our work could also prolong the duration of SARS-CoV-2 immunization.

A recent in vitro study, aimed at investigating the effects of mRNA vaccines encoding for the COVID-19 S protein (mRNA-S) on the fetal–maternal interface, reported that S proteins could interact with ACE-2 receptors, triggering the production of CXCL-10 and CXCL-11 chemokines, IL-6 and IFN-Type 1alpha, molecules that are critical in regulating the immune response in pregnancy. Moreover, the authors observed higher gene expression of the ACE-2 receptor, pivotal for COVID-19 access into host cells [49], in chorionic trophoblast cells compared to other fetal membrane layers [50]. Besides its role in the viral mechanism of infection, ACE-2 activation possesses beneficial downstream effects due to its antioxidant and vasodilatory properties [50]. In accordance with this, our results corroborate these previous findings, adding the placental CAT and SOD enzymatic antioxidant boost to the positive effects of mRNA vaccination during pregnancy.

## 5. Conclusions

In conclusion, our data demonstrate that the placental antioxidant adaptation against COVID-19-induced OxS in placentae is enhanced by the SARS-CoV-2 vaccine, adding further evidence of the role of vaccination as a key and safe tool for the prevention of pregnancy complications.

## Figures and Tables

**Table 1:** Clinical features of vaccinated and unvaccinated COVID-19-positive women and of COVID-19-negative control subgroups. Values are expressed as median ± SE or percentage. Significant differences (*p* < 0.05): * significant compared with v-CTRL; ^ significant compared with u-CTRL; ^§^ significant compared with v-COVID-19; ° significant compared with u-COVID-19.

	v-CTRL (n = 19)	u-CTRL (n = 19)	v-COVID-19 (n = 27)	u-COVID-19 (n = 25)	*p* Value
**Nulliparous (%)**	42.1 (n = 8)	26.3 (n = 5)	33.3 (n = 9)	32 (n = 8)	*p* > 0.05
**Maternal age at delivery (years)**	35.3 ± 1	34.1 ± 1.3	32.8 ± 0.7	31.4 ± 1.4	*p* > 0.05
**Gestational age at delivery (weeks)**	37.5 ± 0.8	38.6 ± 0.3	38.8 ± 0.5	39 ± 0.3	*p* > 0.05
**One vaccine dose (%)**	5.3 (n = 1)	0	11.1 (n = 3)	0	*p* > 0.05
**Two vaccine doses (%)**	42.1 (n = 8)	0 *	59.2 (n = 16) *^	0 ^§^	*^^§^ *p* < 0.001
**Three vaccine doses (%)**	52.6 (n = 10)	0 *	29.6 (n = 8) ^	0 *^§^	* *p* < 0.001
					^ *p* = 0.009
					^§^ *p* = 0.003
**Pre-Pregnancy co-morbidity:**					
Autoimmune disease (%)	10.5 (n = 2)	10.5 (n = 2)	11.1 (n = 3)	12 (n = 3)	*p* > 0.05
Thyroid dysfunction (%)	5.3 (n = 1)	0	3.7 (n = 1)	8 (n = 2)	*p* > 0.05
Uterine myoma (%)	26.3 (n = 5)	5.3 (n = 1)	3.7 (n = 1)	8 (n = 2)	*p* > 0.05
BMI >30 (%)	0	21 (n = 4)	14.8 (n = 4)	24 (n = 6)	*p* > 0.05
**Pregnancy complications:**					
Gestational Hypertension (%)	5.3 (n = 1)	5.3 (n = 1)	7.4 (n = 2)	0	*p* > 0.05
IUGR (%)	5.3 (n = 1)	0	0	0	*p* > 0.05
PE (%)	5.3 (n = 1)	0	0	0	*p* > 0.05
Preterm birth (%)	26.3 (n = 5)	10.5 (n = 2)	3.7 (n = 1)	8 (n = 2)	*p* > 0.05
Cholestasis (%)	0	10.5 (n = 2)	0	8 (n = 2)	*p* > 0.05
GDM (%)	21 (n = 4)	15.8 (n = 3)	18.5 (n = 5)	20 (n = 5)	*p* > 0.05
**SARS-CoV-2 symptoms:**					
Dyspnea (%)	0	0	7.4 (n = 2)	0	*p* > 0.05
Fever (%)	0	0	7.4 (n = 2)	32 (n = 8) *^^§^	*^ *p* = 0.006; ^§^ *p* = 0.025
Anosmia/Ageusia/Asthenia(%)	0	0	0	16 (n = 4) °	° *p* = 0.031
Cough (%)	0	0	11.1 (n = 3)	12 (n = 3)	*p* > 0.05
Rhinitis (%)	0	0	3.7 (n = 1)	16 (n = 4)	*p* > 0.05
**Obstetrics and Neonatal outcomes:**					
Pathological Doppler (%)	0	0	3.7 (n = 1)	4 (n = 1)	*p* > 0.05
Pathological CTG (%)	5.3 (n = 1)	5.3 (n = 1)	14.8 (n = 4)	4 (n = 1)	*p* > 0.05
Fetal Biometry:					
>95th centile (%)	15.8 (n = 3)	15.8 (n = 3)	22.2 (n = 6)	20 (n = 5)	*p* > 0.05
<5th centile (%)	5.3 (n = 1)	5.3 (n = 1)	3.7 (n = 1)	0	*p* > 0.05
					
Caesarean section (%)	52.6 (n = 10)	31.6 (n = 6)	63 (n = 17)	52 (n = 13)	*p* > 0.05
Birth weight (g)	2975 ± 151	3152.6 ± 75	3259.2 ± 124.8	3277 ± 96.5	*p* > 0.05
Placental weight (g)	545.8 ± 22.8	594.6 ± 22.1	535 ± 29.4	576.7 ± 24.1	*p* > 0.05
APGAR < 7 at 5 min (%)	0	0	3.7 (n = 1)	0	*p* > 0.05
Female fetus (%)	57.9 (n = 11)	31.6 (n = 6)	66.7 (n = 18) ^	32 (n = 8) ^§^	^ *p* = 0.019; ^§^ *p* = 0.012
Male fetus (%)	42.1 (n = 8)	68.4 (n = 13)	33.3 (n = 9) ^	68 (n = 17) ^§^	^ *p* = 0.019; ^§^ *p* = 0.012

## Data Availability

The raw data supporting the conclusions of this article will be made available by the authors without undue reservation.
